# A call for Effective Interventions to Curb Shisha Tobacco Smoking among University Students in Eastern Province, Saudi Arabia: Findings from a Cross-Sectional Study

**DOI:** 10.31557/APJCP.2019.20.10.2971

**Published:** 2019

**Authors:** Dhfer Alshayban, Royes Joseph

**Affiliations:** *Department of Pharmacy Practice, College of Clinical Pharmacy, Imam Abdulrahman Bin Faisal University, Dammam-31441, Saudi Arabia. *

**Keywords:** Smoking water pipes, prevalence, knowledge, universities, Saudi Arabia

## Abstract

**Background:**

Although the number of cigarettes smoked has been declining due to major public health initiatives, shisha tobacco smoking is gaining popularity around the world, particularly among youth and university students.

**Methods:**

A cross-sectional study was conducted among 464 university students in Eastern Province of Saudi Arabia using a structured questionnaire (i) to assess the prevalence of shisha smoking; (ii) to evaluate risk-perception, knowledge and normative belief about shisha smoking, and to compare these among users and non-users of shisha.

**Results:**

The study reported a high prevalence (22.8%) of the current use of shisha among the university students with a narrow gender gap and found that STS is getting more popular than other forms of tobacco. A quarter of participants had low risk perception and 30.2% had low knowledge of shisha smoking harm. Importantly, more than two-fifth of them were current users of shisha. Low risk-perception about shisha and social acceptability were significantly contributed to the high prevalence of shisha smoking among the youth.

**Conclusion:**

It is important to provide exposure to education related to shisha hazards and increase the awareness of students and the public about the health effects of shisha smoking.

## Introduction

Tobacco use appears to be increasing in the African and Eastern Mediterranean Regions (World Health Organization, 2015). According to the Tobacco Atlas, more than 20,000 children (10-14 years old) and 3.35 million adults (15+ years old) continue to use tobacco daily in Saudi Arabia, and more than 7,000 deaths occur every year due to tobacco-caused diseases in the country (Drope et al., 2018). Although the number of cigarettes smoked has been declining due to major public health initiatives (Drope et al., 2018), shisha (also referred to as waterpipe, Hookah, Narghile, or Arghile) tobacco smoking (STS) is gaining popularity in the Eastern Mediterranean Region, with no exemption to Saudi Arabia, and around the world, particularly among youth and university students (Maziak, 2011; Naeem, 2011; Akl et al., 2015; Jawad et al., 2018). The latest Saudi Arabia global youth tobacco survey of students aged 13-15 years, which was conducted in 2010, reported that 9.5% of students were current smokers of shisha (Centers for Disease Control and Prevention, 2010). The reports of regional surveys at this period indicated that the prevalence of shisha use ranged from 13%-33% among high school and university students (Amin et al., 2010; Taha et al., 2010; Naeem, 2011; Al Moamary et al., 2012; Jradi and Al-Shehri, 2014; AlSwuailem et al., 2014) in the central and eastern Saudi Arabia. A notable increase in shisha use has been reported recently from the central and the western regions (Awan et al., 2016; Al Ghobain et al., 2018; Daradka et al., 2019), but no such estimate is available from the eastern region of Saudi Arabia.

Studies have been conducted on the risk associated with STS including its health effects, the addictive potential and the cross infection risk of sharing shisha mouthpiece (Eissenberg and Shihadeh, 2009; Aslam et al., 2014; WHO Study Group on Tobacco Product Regulation, 2015; Aboaziza and Eissenberg, 2015; Maziak et al., 2015b; Primack et al., 2016; Waziry et al., 2016). The studies suggest that shisha smokers may inhale a larger volume of smoke than cigarette smokers in a single session, and no or very little nicotine filtered out when the smoke passes through the water-filled chamber of shisha device (Primack et al., 2016). These studies concluded that STS has the health risks that are associated with cigarette smoking, including addiction (Aboaziza and Eissenberg, 2015). Evidence showed that STS is associated with many diseases including lung, gastric and oesophageal cancer, heart and lung diseases, periodontal disease, and low birth weight (Waziry et al., 2016). A cross-sectional study (2015) of male students from a university in the central region of Saudi Arabia reported that knowledge about the health hazards of STS was low among the students (Awan et al., 2016). Studies from neighboring countries of Saudi Arabia, which were conducted among young shisha users, observed that a substantial proportion of the shisha users viewed shisha as a safer alternative to cigarettes (Jaam et al., 2016; Abu-Rmeileh et al., 2018). Lack of effective interventions that target young population, who smoke shisha, is a serious public health concern in Saudi Arabia (Maziak et al., 2015a).

World Health Organization Study Group on Tobacco Product Regulation calls for more research on all aspects of STS including the types and patterns of STS in all regions and cultures (WHO Study Group on Tobacco Product Regulation, 2015). The objectives of this study were (i) to assess the prevalence of STS and its predictors among the university students in Eastern Province of Saudi Arabia; and (ii) to evaluate risk-perception, knowledge and normative belief about STS, and compared these among users and non-users of STS.

## Materials and Methods

*Study design and participant*s

A cross-sectional study was conducted among the undergraduate students during March-May 2018. A minimum sample size of 323 was calculated to estimate a prevalence rate of 30% with the absolute precision of 0.05 and 95% confidence level. We targeted a sample of 500 students from the colleges of Imam Abdulrahman Bin Faisal University (IAU), Saudi Arabia which is one of the leading public universities in the Eastern Region. A group of three final year students from the university were recruited and trained for data collection using the study questionnaire. The group approached other students in public places, such as atrium, café and library, within the colleges, and requested them to fill the questionnaire. The participants were informed about the aim of the study and data collectors’ availability for clarifications on the questionnaire items. Informed consent was obtained from each participant before they participate in the study, and the study was approved by the research and ethics committee of the IAU.

*Questionnaire*

The survey instrument was developed, in Arabic, from a literature review and questions adapted from previously published STS studies (Primack et al., 2008). The first section was about socio-demographics details such as age, gender, study stream, residence location (urban/rural), marital status (single/married), and family monthly income. The second section was intended to assess the prevalence of smoking shisha, cigarettes and e-cigarettes. The participants were asked if they had smoked these forms of tobacco within the past one year (for 12-month prevalence) and within the past 30 days (for 30-day prevalence). They were also asked to report how frequent their family members or friends use STS. 

The third section consisted of two questions related to risk-perception about STS where the participants were asked to rate harmfulness and addictiveness of STS compared to cigarettes on a five-point scale ranging from ‘STS is much less harmful/addictive’ with score of ‘0’to STS is much more harmful/addictive with score of ‘4’. A summary risk-perception score was calculated by adding these scores, and it ranges from 0 to 8 with the low score represents high false perception about STS. The next section included five items related to knowledge of STS-related hazards: the level of knowledge was assessed based on the ability of students to recognize the toxic contents of STS (water filtering of toxins; absence of tar, nicotine and carbon monoxide) and to identify diseases associated with STS (no increased risk of cardiovascular diseases [CVD]). The responses to these items were obtained on a 5-point Likert scale with score ranges from 0 for strongly agree to 4 for strongly disagree. A summary knowledge score was computed, and the summative score can be ranged from 0 to 20 with the higher the score represents the higher knowledge about the health effects of STS. Finally, to measure the normative beliefs, the participants were asked to rate the social acceptance of STS in the society. The response scale included “not acceptable”, “somewhat acceptable”, “moderately acceptable”, and “very acceptable”.

**Table 1:** Socio-Semographics and Shisha Tobacco Smoking (N=464)

Factors	Total	Shisha used within 30 days	
	n (%)	n (%)	p-value^1^
Gender			
Male	268 (57.8%)	68 (25.4%)	0.129
Female	196 (42.2%)	38 (19.4%)	
Area of study			
Health	300 (64.7%)	70 (23.3%)	0.895
Engineering	78 (16.8%)	18 (23.1%)	
Arts, Science and Management	86 (18.5%)	18 (20.9%)	
Locality			
Urban	378 (81.5%)	98 (25.9%)	0.001*
Rural	86 (18.5%)	8 (9.3%)	
Marital status			
Single	436 (94%)	100 (22.9%)	0.854
Married	28 (6%)	6 (21.4%)	
Family monthly income			
Less than 5,000 SR	74 (15.9%)	12 (16.2%)	0.063
5,000-15,000	142 (30.6%)	24 (16.9%)	
16,000-25,000	154 (33.2%)	48 (31.2%)	
Greater than 25,000	94 (20.3%)	22 (23.4%)	

^1^Chi-square test was carried out; *statistically significant

**Table 2:** Cigarette Smoking and Shisha Tobacco Smoking (N=464)

Factors	Total	Shisha used within 30 days	
	n (%)	n (%)	p-value^1^
Cigarettes used within 12 months			
No	352 (75.9%)	42 (11.9%)	<0.001*
Yes	112 (24.1%)	64 (57.1%)	
Cigarettes used within 30 days			
No	362 (78%)	46 (12.7%)	<0.001*
Yes	102 (22%)	60 (58.8%)	
e-cigarettes used within 12 months			
No	360 (77.6%)	48 (13.3%)	<0.001*
Yes	104 (22.4%)	58 (55.8%)	
e-cigarettes used within 30 days			
No	392 (84.5%)	62 (15.8%)	<0.001*
Yes	72 (15.5%)	44 (61.1%)	
Use of Shisha by family/friends			
Never used	168 (36.2%)	16 (9.5%)	<0.001*
Rarely used	94 (20.3%)	18 (19.1%)	
Occasionally used	90 (19.4%)	26 (28.9%)	
Frequently used	112 (24.1%)	46 (41.1%)	

^1^Chi-square test was carried out; *statistically significant

**Table 3:** Prevalence of Past 30-Day STS, and STS-Related Risk-Perception, Knowledge and Normative Beliefs among the University Students (N=464)

Factors	Total	Shisha used within 30 days	
	n (%)	n (%)	p-value
Belief about harmfulness compared to Cigarettes			
Shisha is more harmful	140 (30.2)	20 (14.3)	<0.001*
Shisha is equally harmful	146 (31.5)	20 (13.7)	
Shisha is less harmful	178 (38.4)	66 (37.1)	
Belief about addictiveness			
Shisha is more Addictive	152 (32.8)	22 (14.5)	<0.001*
Shisha is equally Addictive	108 (23.3)	14 (13.0)	
Shisha is less Addictive	204 (44.0)	70 (34.3)	
Risk-perception score (overall)			
Low (highly favoured for shisha)	116 (25.0)	50 (43.1)	<0.001*
Moderate	238 (51.3)	48 (20.2)	
High (Less favoured for shisha)	110 (23.7)	8 (7.3)	
Belief about water filtering of toxins			
Completely‎/Substantially	120 (25.9)	48 (40.0)	<0.001*
Moderately	218 (47.0)	46 (21.1)	
Slightly‎/Nothing	126 (27.2)	12 (9.5)	
Belief about absence of tar			
Agree	240 (51.7)	72 (30)	<0.001*
Neutral	70 (15.1)	16 (22.9)	
Disagree	154 (33.2)	18 (11.7)	
Belief about absence of nicotine			
Agree	204 (44.0)	64 (31.4)	<0.001*
Neutral	80 (17.2)	26 (32.5)	
Disagree	180 (38.8)	16 (8.9)	
Belief about absence of carbon monoxide			
Agree	210 (45.3)	68 (32.4)	<0.001*
Neutral	46 (9.9)	14 (30.4)	
Disagree	208 (44.8)	24 (11.5)	
Belief about increased risk of CVD			
Disagree	132 (28.4)	62 (47)	<0.001
Neutral	72 (15.5)	18 (25)	
Agree	260 (56)	26 (10)	
Knowledge score (overall)			
Low (highly favoured for shisha)	140 (30.2)	58 (41.4)	<0.001
Moderate	170 (36.6)	32 (18.8)	
High (Less favoured for shisha)	154 (33.2)	16 (10.4)	
Normative belief (Social acceptance)			
Not acceptable	146 (31.5)	14 (9.6)	<0.001*
Somewhat acceptable	152 (32.8)	32 (21.1)	
Moderately acceptable	100 (21.6)	34 (34.0)	
Very acceptable	66 (14.2)	26 (39.4)	

^1^Chi-square test was carried out; *statistically significant

**Table 4:** Multivariable Logistic Model for 30-day STS (N=464)

Factors	AOR (95% CI)	p-value
Locality		
Urban	6.28 (2.11-18.69)	0.001*
Rural	Reference	
Cigarettes used within 12 months		
No	Reference	
Yes	5.44 (2.94-10.05)	<0.001*
e-cigarettes used within 12 months		
No	Reference	
Yes	4.86 (2.57-9.21)	0.001*
Use of Shisha by family		
Never used	Reference	
Rarely used	1.91 (0.8-4.58)	0.146
Occasionally used	2.78 (1.13-6.81)	0.025*
Frequently used	2.97 (1.32-6.68)	0.008*
Risk-perception score (overall)		
Low (highly favoured for shisha)	6.89 (1.94-24.47)	0.003*
Moderate	2.21 (0.72-6.82)	0.169
High (Less favoured for shisha)	Reference	
Knowledge score (overall)		
Low (highly favoured for shisha)	1.43 (0.55-3.72)	0.464
Moderate	0.83 (0.33-2.13)	0.702
High (Less favoured for shisha)	Reference	
Normative belief (Social acceptance)		
Not acceptable	Reference	
Somewhat acceptable	2.6 (1.1-6.14)	0.029*
Moderately acceptable	4.9 (2.05-11.73)	<0.001*
Very acceptable	5.48 (2.1-14.28)	0.001*

AOR, adjusted odds ratio; CI, Confidence interval; *statistically significant

**Figure 1:**
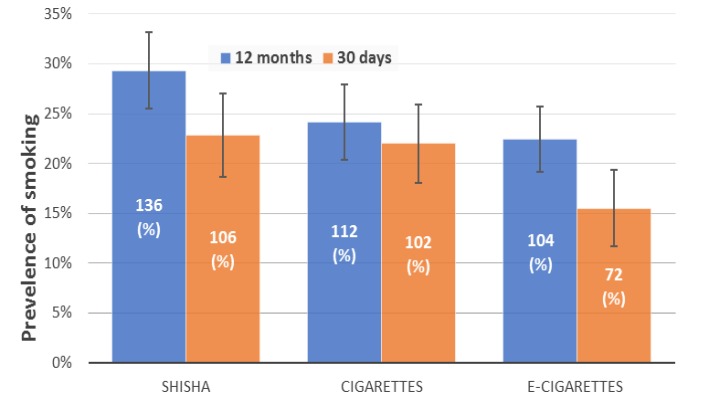
Past 12-month and 30-day Prevalence of Smoking. The vertical line represents the 95% confidence interval

*Analysis*

The past 30-day and past 12-month users were defined as those who smoked at least one time in the previous 30 days and previous 12 months respectively. Past 30-day use of STS was considered the primary outcome. The responses to items in risk-perception and knowledge factors were collapsed into three categories for the analysis purpose. Additionally, the risk-perception and knowledge scores were also collapsed into tertiles, which represent the categories low score (highly favored for shisha), moderate score and high score (less favored for shisha). 

Data were presented as frequencies and percentages. Univariate analysis using chi-square statistics was initially carried out test the association between past 30-day use of STS and predictor variables. Predicator variables include socio-demographic characteristics, smoking behavior, risk-perception score, knowledge score, and social acceptance rating. Odds ratios and 95% confidence intervals were then derived using multiple logistic regression, adjusting the predictor variables that found statistically significant in the univariate analysis. For all analyses, a p-value less than 0.05 (two-sided) indicated statistical significance. Statistical analyses were conducted using SPSS Statistics (version 24.0).

## Results

*Socio-demographic information*

The study questionnaires were filled by 464 university students with a response rate of 92.8%. Table 1, in the second column, summarizes the socio-demographic characteristics of participants. The mean (SD) age was 20.7 (1.3) years, and 64.7% of participants were from health stream. The sample comprised of more male students, and with equal representation of participants from different income levels.

*Prevalence of smoking: shisha, cigarettes, and e-cigarettes*

Figure 1 presents the past 12-month and 30-day prevalence (with 95% confidence interval) of smoking shisha, cigarettes and e-cigarettes. The self-reported past 12-month and 30-day prevalence of STS were 29.3% and 22.8%, respectively. The prevalence of shisha smoking was slightly higher than that of cigarettes smoking and noticeably higher than that of e-cigarettes smoking among the participants.

Table 1 presents the past 30-day STS status among the socio-demographic levels. Importantly, no significant difference in prevalence of STS between male and female students was observed, though the proportion was slightly higher among male students (25.4% vs. 19.4%; p-value=0.129). The table shows that urban students were more prone to STS compared to rural students (25.9% vs. 9.3%; p-value=0.001). There was no difference in prevalence of STS between levels of study stream, marital status, or family income (p-value>0.05). 

Table 2 presents the past 30-day STS status among users of cigarette and e-cigarette. The past 30-day shisha use was significantly associated with the cigarette/e-cigarette use and the use of shisha among the family members or friends (p-value<0.001).

*STS-related risk-perception, knowledge and normative beliefs*

Table 3 presents STS-related risk-perception, knowledge and normative beliefs among the participants, in overall and by past 30-day STS status. 

Regarding the risk-perception, 38.4% and 44.0% of participants answered that STS is less harmful and less addictive, respectively, compared to cigarette smoking. Importantly, significantly higher past 30-day STS prevalence was reported among these groups compared to others. Thus, in combined, 43.1% of participants with low risk-perception score were shisha user in the past 30 days compared to the prevalence of 7.3% among participants with a high risk-perception score (p-value<0.001). 

Regarding the STS-related knowledge, a substantial proportion of participants believed that water filters toxins substantially or completely (25.9%), STS is free of tar (51.7%), nicotine (44.0%) or carbon monoxide (45.3%), and disagreed with the increased risk of CVD (28.4%). A significant proportion (30.0%–47.0%) of them had used shisha in the past 30 days. Thus, in overall, 41.4% of participants with low knowledge score were shisha user in the past 30 days compared to the prevalence of 10.4% among participants with high knowledge score (p-value<0.001).

Of the sample, 35.8% considered STS as “moderately-very socially acceptable”. The prevalence of STS was significantly higher in these groups (34.0%–39.4%) compared to the contrary groups (9.6%–21.1%; p-value<0.001).

*Multivariable model*

In a fully adjusted multivariable logistic model (Table 4), past 30-day STS was significantly associated with both cigarette and e-cigarette use, urban status, low perceived risk, and belief about the social acceptance. The level of knowledge was not significantly associated with the current use of STS after adjusted for other factors, particularly the level of risk-perception.

## Discussion

The present cross-sectional study assessed the prevalence of STS among university students based on a sample from the colleges of a leading university in Eastern Region, Saudi Arabia. The study also evaluated risk-perception, knowledge and normative belief about STS among the university students, and compared these among users and non-users of STS. Our study shows that the past 30-day and 12-month prevalence of STS among university students were 22.8% and 29.3%, respectively. The prevalence of STS from the study was substantially higher than the prevalence of 12.6% which had reported in 2008 among a sample from the same university (Taha et al., 2010); the finding indicates a noticeable increase in shisha use over the time. Similarly, a high prevalence of STS among students was also reported from universities in Al Madinah and Riyadh regions of Saudi Arabia (Awan et al., 2016; Daradka et al., 2019), in other countries in the Eastern Mediterranean Region (Roohafza et al., 2015; Jaam et al., 2016; Tucktuck et al., 2018). The current estimate was also higher than that among the general adult population in Saudi Arabia (Al Nomay and E Ahmed, 2015). A recent study among physicians in Riyadh, Saudi Arabia showed a high prevalence of past 30-day use of STS (45%), and the highest prevalence was reported among newly graduated trainees (Al Ghobain et al., 2018). The availability of flavored tobacco, the thriving coffee shop culture and the limited regulations boost the spread of STS globally (Maziak et al., 2015b). The findings reflect an emerging STS epidemic among youth in Saudi Arabia and other countries in the Eastern Mediterranean Region.

Although the prevalence of cigarette smoking was relatively higher among males in Saudi Arabia (Algabbani et al., 2018), our study reported a high prevalence of STS among males and female students with a narrow gender gap in consistent with a recent study from the western region of Saudi Arabia (Daradka et al., 2019). A national survey on tobacco use among individuals aged 15 years or older in Saudi Arabia also reported a substantially lower female-male ratio on the prevalence of daily STS compared to that of daily use of all forms of tobacco (Moradi-Lakeh et al., 2015). 

Importantly, along with other studies in the Eastern Mediterranean Region (Roohafza et al., 2015; Tucktuck et al., 2018), our study found that the prevalence of STS had surpassed that of cigarette or e-cigarette smoking. The recent study from the western region of Saudi Arabia reported that the prevalence of shisha smoking did not relatively differ from that of cigarettes smoking (Daradka et al., 2019). Further, the present study observed that more than half of the current cigarette or e-cigarette smokers were current shisha smokers, whereas, no more than 16% of non-users of other forms of tobacco were current shisha smokers. These findings indicate that STS has increasing popularity and is getting more common than cigarettes or e-cigarettes smoking among youth in both genders. The current study also found that substantially higher prevalence of STS among the participants whose close family members or friends were frequent users of shisha or the participants who believed STS is socially well acceptable. The findings support an argument that socio-cultural norms towards STS far outweighed its health impacts (Afifi et al., 2013).

Many people have a misconception that STS is a safer alternative to cigarette smoking. A recent study that had been conducted among the ever users of STS in the five neighboring countries reported that 44% of users believed STS is less addictive than cigarette smoking with the highest of 54% among Egyptian users (Abu-Rmeileh et al., 2018); in our study, this estimate was 66% of past-30 day users. A similar pattern was observed regarding the belief about the harmfulness of STS. In overall, according to the multiple logistic model in the current study, those with low risk-perception about STS had more than seven times the odds of being a past 30-day shisha user compared with those with high risk-perception about STS. Despite of evidence for the harmful and addictive effects (Aslam et al., 2014; Aboaziza and Eissenberg, 2015; Primack et al., 2016), STS is often used as a safe alternative of cigarette smoking probably due to the misconception.

The misconception about the harmful and addictive effects of STS is linked with the lack of awareness of the health effects of STS (Salloum et al., 2017). Researchers have demonstrated that one session of STS was associated with more than 100 times volume of smoke inhalation and higher levels of nicotine, tar and carbon monoxide compared with one cigarette, in contrast to the misconception that water used in the pipe absorbs toxic elements (Primack et al., 2016). In the current study, nearly three-fourths of the participating students believed that the water used in the pipe moderately or substantially removes the toxic elements. In particular, 52%, 44% and 45% believed STS to be safe from tar, nicotine and carbon monoxide respectively. Further, 28% were failed to recognize STS as a risk factor for CVD. Importantly, a substantial proportion of these students were current shisha users. The multivariate model showed that the overall knowledge score was not a significant independent predictor of current shisha use, whereas the risk-perception was a significant predictor. Studies have reported that knowledge is positively associated with risk-perception (Allen and Butler, 1993). Thus, the ignorance of the health effects of STS is a major concern, and it is important to provide exposure to education related to STS hazards and increase the awareness of students and public about the health effects of STS.

Some limitations should be noted. The study was conducted among a convenient sample of undergraduate students from a university in the Eastern Province, Saudi Arabia. However, the selection of the sample from different colleges within the university and the large sample size ensured fair representation of students from diverse socio-economic backgrounds. The study could have overestimated the prevalence of STS due to data collection at public places within the university where a number of students might have keen interest in social gatherings.

In conclusion, the current study reported a high prevalence of current use of shisha among university students with a narrow gender gap, and STS is getting more popular than other forms of tobacco. Importantly, a substantial proportion of the students had low knowledge of STS harm with misconceptions about the harmful and addictive effects of STS, and a significant proportion of these students were current users of STS. Low risk-perception about STS and social acceptability were significantly contributed to the higher prevalence of STS among the youth. The findings of this study indicate an urgent need to promote multi-disciplinary health education activities for increasing awareness among different age groups in order to prevent youth from all type of smoking including shisha. Apart from creating the educational interventions, the Saudi higher authorities should have review and update regulations (i) to issue licenses for cafes and restaurants offering shisha, (ii) to replace misleading labels or descriptions such as ‘100% natural’ with health warning labels on shisha devices/products, and (iii) to limit the availability of flavoured tobacco products.
